# Naturally Circulating Hepatitis A Virus in Olive Baboons, Uganda

**DOI:** 10.3201/eid2207.151837

**Published:** 2016-07

**Authors:** Andrew J. Bennett, Samuel D. Sibley, Michael Lauck, Geoffrey Weny, David Hyeroba, Alex Tumukunde, Thomas C. Friedrich, David H. O’Connor, Caley A. Johnson, Jessica M. Rothman, Tony L. Goldberg

**Affiliations:** University of Wisconsin, Madison, Wisconsin, USA (A.J. Bennett, S.D. Sibley, T.L. Goldberg);; Wisconsin National Primate Research Center, Madison (M. Lauck, T.C. Friedrich, D.H. O’Connor, T.L. Goldberg);; Makerere University, Kampala, Uganda (G. Weny, D. Hyeroba, A. Tumukunde, J.M. Rothman, T.L. Goldberg);; Graduate Center of City University of New York, New York, New York, USA (C.A. Johnson, J.M. Rothman);; New York Consortium in Evolutionary Primatology, New York (C.A. Johnson, J.M. Rothman);; Hunter College of the City University of New York, New York (J. Rothman).

**Keywords:** Hepatitis A virus, picornavirus, metagenomics, olive baboon, hepatovirus, deep sequencing, primate, viruses, zoonoses, Uganda

**To the Editor:** Hepatitis A (HAV; family *Picornaviridae*; genus *Hepatovirus*) is an ≈7.5-kb single-stranded positive-sense RNA virus that causes acute inflammation of the liver in humans and nonhuman primates. Although HAV is most commonly transmitted by food and water contaminated with feces, humans have acquired HAV from handling infected nonhuman primates in captivity (*1*). 

HAV has been detected in recently imported captive primates after spontaneous outbreaks of acute hepatitis in animal facilities, but the definitive hosts of this virus have remained obscure (*2*,*3*). We identified by next-generation sequencing HAV in the blood of a free-living olive baboon (*Papio anubis*) from Kibale National Park, Uganda, sampled in September 2010. Subsequent testing of a separate Kibale olive baboon troop in 2014 indicated the virus was prevalent and shed in feces.

As part of a long-term study of nonhuman primate health and ecology, 23 animals were immobilized and sampled in 2010 as previously described (*4*). All animal protocols received prior approval from the Uganda National Council for Science and Technology (Kampala, Uganda), the Uganda Wildlife Authority (Kampala, Uganda), and the University of Wisconsin–Madison Animal Care and Use Committee (Madison, WI, USA). All samples were shipped in accordance with international laws under Convention on International Trade in Endangered Species of Wild Fauna and Flora Ugandan permit no. 002290.

During May 2012, we subjected total RNA from 1 mL of blood plasma of each animal to next-generation sequencing as previously described (*4*); results showed HAV-like sequences in 1 of 23 baboons. De novo assembly of these reads yielded a nearly complete HAV genome, which we term KibOB-1. KibOB-1 is most similar (94.2% nt identity; Figure) to AGM-27, an HAV originally detected in an African green monkey (*Chlorocebus aethiops*) imported to a Russian primate facility from Kenya (*3*).

**Figure:**
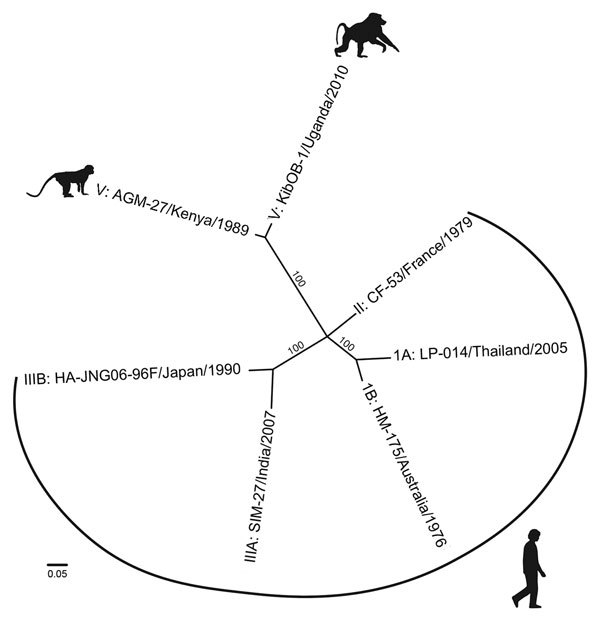
Whole-genome phylogenetic reconstruction of representative HAVs. HAVs are grouped into 6 genotypes based on 168 bp of the C-terminal extension of the viral protein 1 gene. Baboon HAV detected in Kibale National Park, Uganda, in 2010 and 2014 (GenBank accession number KT819575) clusters with AGM-27 (*3*), previously the sole member of genotype V. jModeltest 2 (http://jmodeltest.org) was used to find the best-fit evolutionary model for the data, after which the maximum-likelihood tree was estimated using the heuristic search method in PAUP* (http://paup.csit.fsu.edu), with starting trees obtained by neighbor-joining, random stepwise addition, and branch swapping by tree-bisection reconnection and starting branch lengths obtained using Rogers-Swofford approximation. Bootstrap values were derived from 1,000 replicates of the heuristic search; only values >50% are shown. GenBank accession nos.: IA, EF207320; IB, M14707; II, AY644676; IIIA, FJ227135; IIIB, AB258387; V, D00924). HAV, hepatitis A virus. Scale bar indicates substitutions per site.

For 11 baboons, we also collected a paired fecal sample, which we analyzed for evidence of viral shedding. Samples were preserved in RNAlater (Ambion Inc., Austin, TX, USA) at −20°C, and viral RNA was isolated by using the ZR Soil/Fecal RNA Microprep kit (Zymo Research, Irvine, CA, USA) following manufacturer’s protocols. Reverse transcription PCR (RT-PCR) of RNA was primed with random hexamers by using the RNA to cDNA Ecodry Premix (Random Hexamers) (Clontech Laboratories, Inc., Mountain View, CA, USA), and diagnostic PCR was conducted with primers flanking the C-terminal extension of the HAV viral protein (VP) 1 gene (*pX*) by using the High Fidelity PCR Master Mix-Ecodry Premix (Clontech Laboratories, Inc.). Five of 11 paired fecal samples tested positive for HAV by RT-PCR, indicating a higher prevalence of the virus in feces than in blood.

We then surveyed a second troop of habituated olive baboons at the same field site during February–April 2014 (*5*). From these baboons, 7 of 19 fecal samples tested positive by RT-PCR. Confirmatory Sanger sequencing of RT-PCR amplicons was successful for 3 of these 7 animals (GenBank accession nos. KT819576–KT819578). Phylogenetic analyses of these sequences demonstrate monophyly and a low degree of interhost variability (>94% nt identity).

The risk to humans posed by KibOb-1 remains unknown. Although human infection with HAV genotype V has not been reported, evidence suggests that HAV variants might be capable of infecting a diversity of primate hosts (*6*). Although it is not known whether the closely related AGM-27 strain was discovered infecting its natural host, the similarity of KibOB-1 and AGM-27 raises the possibility of a recent host transfer. Major host shifts characterize the evolutionary histories of recently discovered bat and rodent hepatoviruses (*7*). Host fidelity of KibOB-1 is similarly unknown, but experimental infection of several nonhuman primate species with the similar AGM-27 virus found varying pathogenicity in different species (*6*). In particular, the AGM-27 caused productive infection in chimpanzees, with stimulation of a broadly reactive HAV immunoglobulin response (*6*).

Human and simian HAVs are considered a single serotype (*6*); thus, serosurveillance for HAV in humans might be unable to distinguish between human and zoonotic simian HAV infection, enabling the possibility of cryptic zoonotic transmission. Similarly, detection of HAV antibodies in wild primates, such as in a recent study of baboons in South Africa living in close proximity to humans (*8*), might not indicate anthroponotic transmission of human viruses but rather infection with an endemic HAV.

Prior studies have documented cross-species transmission between the primates of Kibale National Park and neighboring human populations, especially of gastrointestinal pathogens (*9*). A study tracking food-crop–raiding events on 97 farms within 0.5 km of Kibale’s forest edge found that 72% of households faced baboon raids over a 23-month period, including 228 discrete baboon raids (*10*). This finding suggests that a major portion of the local community remains at risk for exposure to potentially infectious baboon excreta. Such exposure, in addition to the evidence presented here that HAV is prevalent in wild baboons of Uganda and is shed into the environment, merits increased attention to the zoonotic risk for simian hepatoviruses.
